# First report of the zoonotic nematode *Baylisascaris procyonis* in non-native raccoons (*Procyon lotor*) from Italy

**DOI:** 10.1186/s13071-021-05116-3

**Published:** 2022-01-12

**Authors:** Andrea Lombardo, Giuseppina Brocherel, Carla Donnini, Gianluca Fichi, Alessia Mariacher, Elena Lavinia Diaconu, Virginia Carfora, Antonio Battisti, Nadia Cappai, Luca Mattioli, Claudio De Liberato

**Affiliations:** 1 Istituto Zooprofilattico Sperimentale del Lazio e Della Toscana “M. Aleandri”, Arezzo, Italy; 2 Istituto Zooprofilattico Sperimentale del Lazio e Della Toscana “M. Aleandri”, Grosseto, Italy; 3 Istituto Zooprofilattico Sperimentale del Lazio e Della Toscana “M. Aleandri”, Rome, Italy; 4 Parco Nazionale Foreste Casentinesi, Monte Falterona e Campigna, Arezzo, Italy; 5 Regione Toscana, Arezzo, Italy

**Keywords:** *Baylisascaris procyonis*, Italy, *Larva migrans*, *Procyon lotor*, Raccoon

## Abstract

*Baylisascaris procyonis* is a nematode parasite of the raccoon (*Procyon lotor*), and it can be responsible for a severe form of *larva migrans* in humans. This parasite has been reported from many countries all over the world, after translocation of its natural host outside its native geographic range, North America. In the period between January and August 2021, 21 raccoons were cage-trapped and euthanized in Tuscany (Central Italy), in the context of a plan aimed at eradicating a reproductive population of this non-native species. All the animals were submitted for necroscopic examination. Adult ascariids were found in the small intestine of seven raccoons (prevalence 33.3%). Parasites have been identified as *B. procyonis* based on both morphometric and molecular approaches. The aim of the present article is to report the first finding of this zoonotic parasite from Italy, highlighting the sanitary risks linked to the introduction of alien vertebrate species in new areas.

**Supplementary Information:**

The online version contains supplementary material available at 10.1186/s13071-021-05116-3.

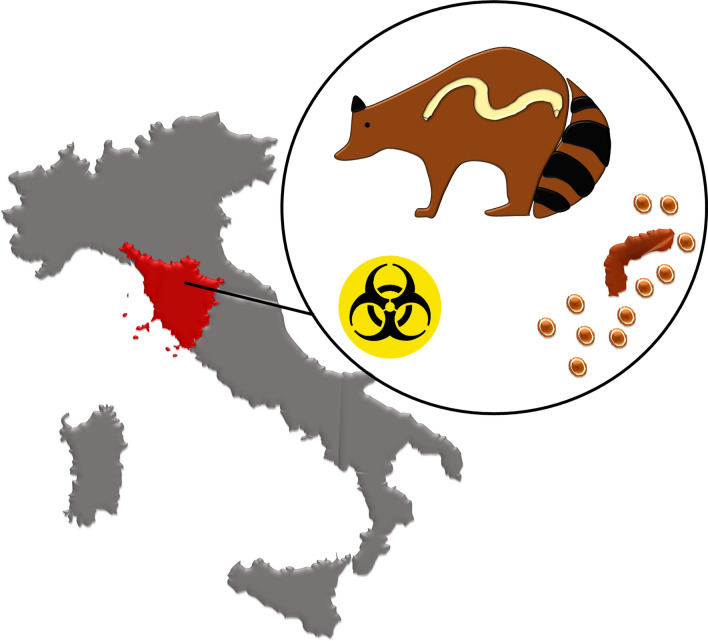

*Baylisascaris procyonis* is an ascariid nematode parasite of the raccoon, *Procyon lotor*. In the native range of its host, North America, its prevalence can be very high, at times exceeding 70% [1, 2]. *Baylisascaris procyonis* has a direct life cycle, following the fecal–oral route. An infected raccoon can shed each day millions of *B. procyonis* eggs, which can remain infective in the environment for years, leading to long-lasting contamination of the habitat [3]. Moreover, many species of paratenic hosts, both birds and mammals, can acquire the infection by ingesting the eggs shed in the environment, the same being for humans [2]. In paratenic hosts, *B. procyonis* larvae migrate to several tissues, including lungs and abdominal viscera (*visceral larva migrans*, VLM), eye (*ocular larva migrans*, OLM), and central nervous system (*neural larva migrans*, NLM), representing a source of infection for raccoons feeding on the paratenic hosts [1]. In human patients, *B. procyonis* larvae are not markedly neurotropic, since a relatively low percentage of larvae actually reach the central nervous system (CNS) [4]. Therefore, infections sustained by a low number of larvae may only elicit seroconversion with subtle CNS disease. Nevertheless, when a higher burden of infective eggs is ingested, massive larval migration can result in CNS colonization, with a very severe or even fatal condition. Most NLM cases in humans involve children, due to pica and poor hygienic behavior [1, 4, 5].

Following the introduction of the raccoon in many European countries, since the 1830s [2] *B. procyonis* has progressively increased its geographical range. At present, this parasite has been reported in non-native raccoon populations from Denmark [6], Austria [7], Poland [8], and Germany [9], where some human infections have been described as well [10, 11]. In Italy, the raccoon was first reported in 2004 in Lombardy, Northern Italy [12]. Nowadays, this species is also present with a reproducing population in Tuscany, Central Italy [13], within the Casentino valley territory. The Casentino valley encompasses the river basin of the upper Arno, within the province of Arezzo, and is characterized by woodlands and hills, interspersed with high-yield crops such as vineyards, fruit trees, white spruce, and black pine. The origin of the raccoon population in Casentino is still debated, but it can probably be traced back to accidental escapes from captivity in the early 2000s. Raccoons have rapidly expanded in the Casentino valley, and some animals have been recently sighted near the Emilia–Romagna border. Due to the invasive nature of the raccoon, and to the environmental and health issues linked to its presence, the Italian authorities set up an eradication plan [14], with the aim of removing raccoon populations in accordance with Regulation (EU) 1143/2014.

The aim of the present article is to describe the first report of *B. procyonis* from non-native raccoons in Italy, emphasizing the sanitary risks linked to the introduction of this species in new areas.

Between January and August 2021, 21 free-ranging adult raccoons (13 males and eight females, weight 4.3 ± 1.9 kg) from the Casentino valley, Arezzo, Tuscany (Central Italy), were cage-trapped in four different sites (Fig. 1) and euthanized in the context of a state-instituted eradication plan. Euthanasia was performed by trained personnel of the local police authority, by shooting with a free bullet*,* according to Reg. 2010/63/EU Annex IV guidelines for “other carnivores.” Trapping, handling, and euthanasia procedures were performed in order to minimize pain, suffering, distress, or lasting harm. The animals were submitted to the authors’ institution for necropsy. In April 2021, on examination of the alimentary tract, one individual was found to harbor ascariid nematodes in the small intestine. After this first finding, six other raccoons were found to harbor these parasites (from one to over 100 adult specimens). In two of the infected raccoons, a partial intestinal obstruction was observed, caused by the high burden of adult parasites (over 100 specimens). Both male and female parasites were recovered. No other parasite species were recovered from the examined animals. The range of parasite burden for each positive raccoon is shown in Table 1.

**Fig. 1:**
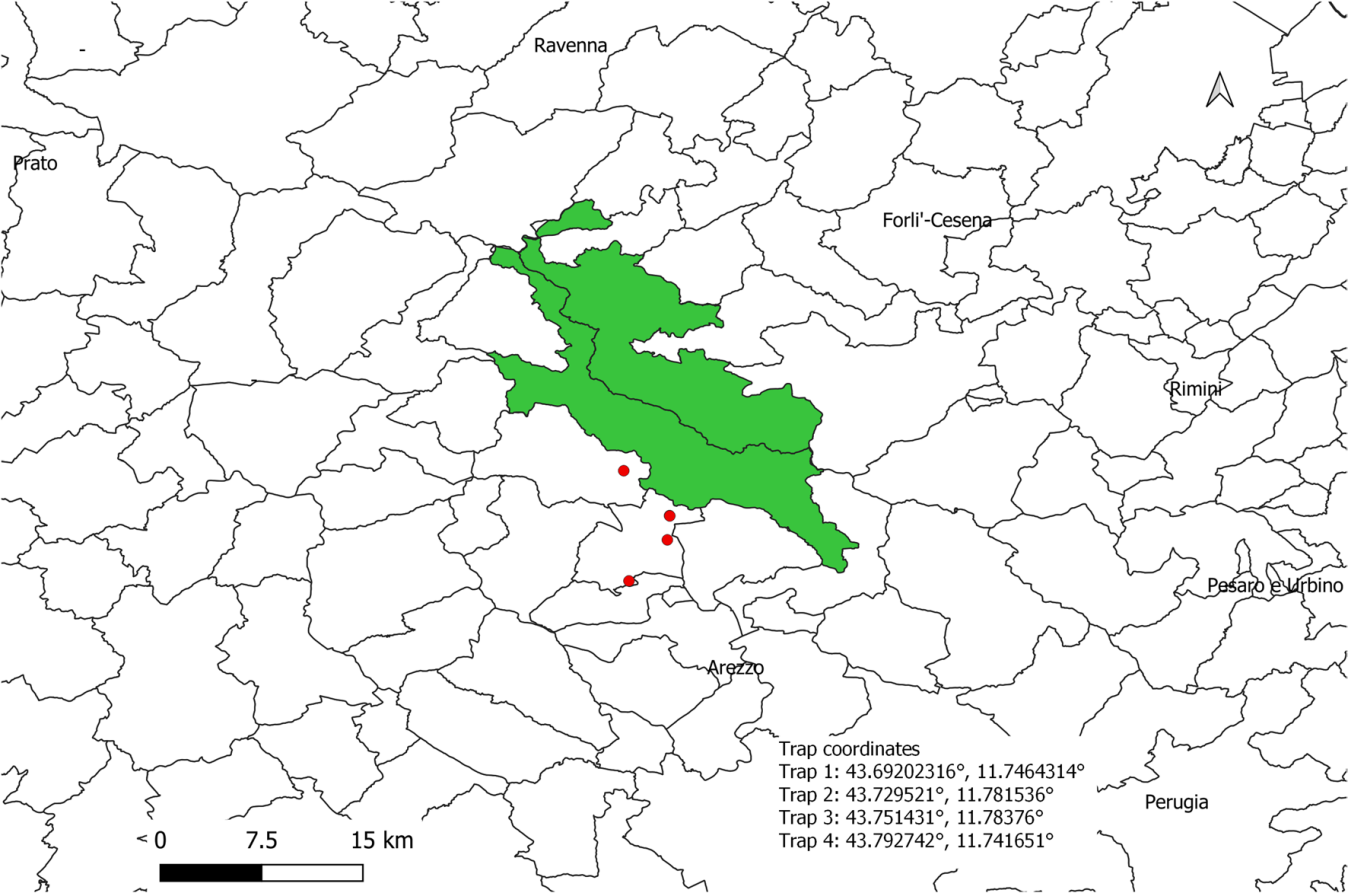
Location of four trapping sites in the Arezzo province, Tuscany, Central Italy, 2021

**Table 1:** Parasitic load in *Baylisascaris procyonis*-positive raccoons

*B. procyonis-*positive raccoons	Number of adult parasites (range)	Presence of intestinal obstruction
1	1–10	No
2	1–10	No
3	20–30	No
4	1–10	No
5	30–40	No
6	> 100	Yes
7	> 100	Yes

Morphometric features were consistent with those of *B. procyonis* as reported by previous studies (Table 2): length 9–16 cm, width 1-3 mm, white-yellowish to tan-colored, with evident black alimentary tract (Additional file 1: Figure S1, Additional file 2: Figure S2). Microscopically, characteristic features were identified (Fig. 2), such as the presence of cervical alae with cuticular bars and a pericloacal *area rugosa* in the males; dorsal and subventral labial papillae distinctly double; stout, uniform spicules and discrete precloacal and postcloacal groups of papillae on the tail in males [5].

**Table 2:** Selected features and respective metrics of *Baylisascaris procyonis*

Features of *B. procyonis*	Measure	References
Adult female length	20–22 cm	[5, 25]
	20 cm	[26]
Adult male length	9–11 cm	[5, 25]
	9 cm	[26]
Male spicules	Usually < 1 mm	[26]
Eggs	Average: 68–76 × 55–61 μm	[5]
	Average: 65–79 μm	[26]
	Range: 63–88 × 50–70 μm	[4, 25]

**Fig. 2:**
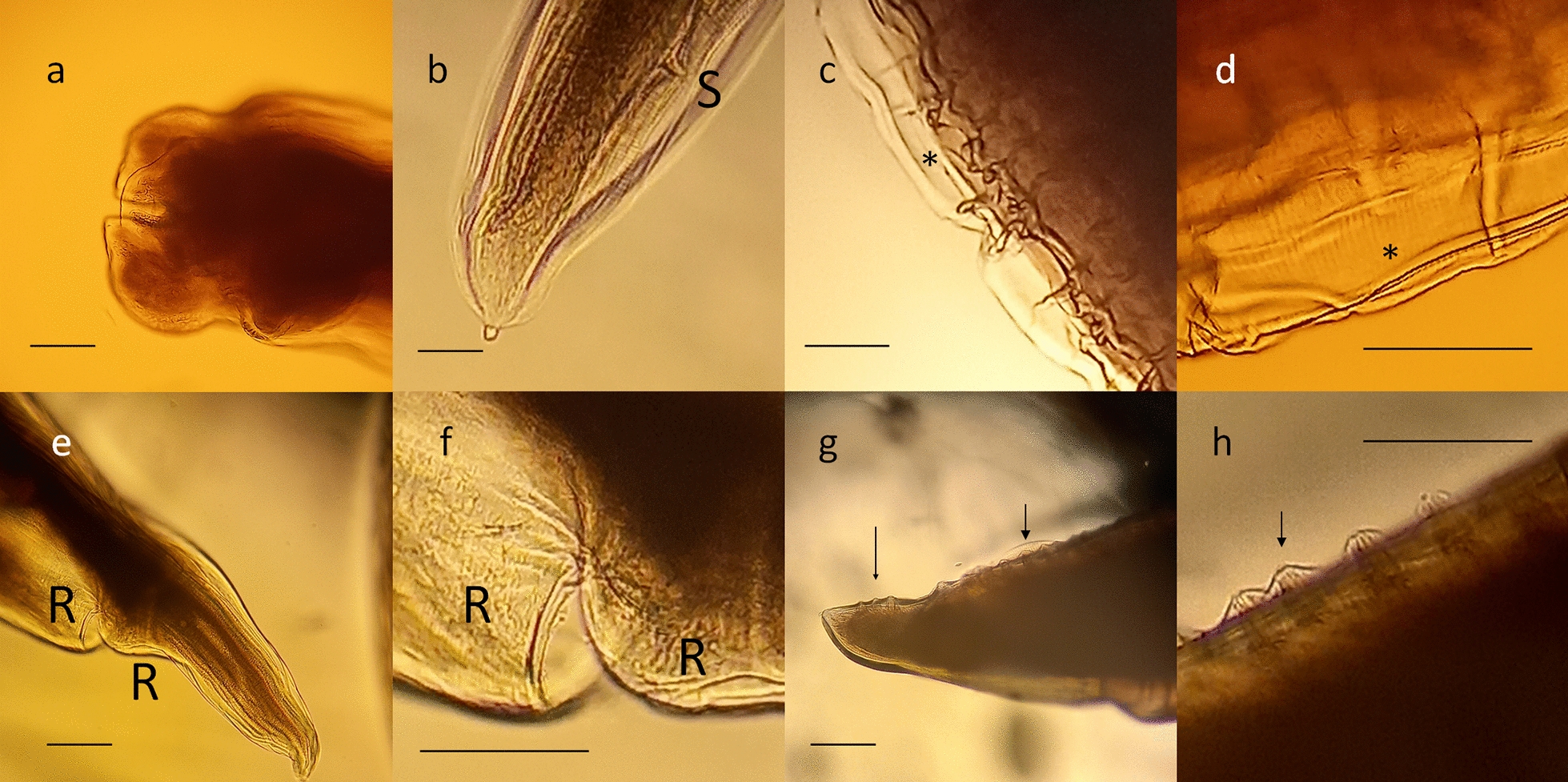
Morphological details of *Baylisascaris procyonis* adult male. **a** Dorsal and subventral labial papillae. **b** Spike-shaped posterior end of the tail and spicule (S). **c**, **d** Cervical alae (*) with cuticular bars. **e**, **f** Pericloacal roughened patches (R). **g** Precloacal (large arrow) and postcloacal groups (thin arrow) of papillae. **h** Precloacal (large arrow) papillae. Scale bar: 10 μm

Identification was confirmed by molecular methods, using a next-generation sequencing (NGS)-based amplicon sequencing approach. Small pieces (approximately one centimeter) of seven ascariid parasites harbored by the positive necropsied raccoons (one parasite for each animal) were completely lysed, following a lysis step with proteinase K. Then, total DNA was extracted using the DNeasy Blood & Tissue Kit (Qiagen, Hilden, Germany), using the manufacturer’s protocol. Amplicon sequencing targeting the internal transcribed spacer 2 (ITS-2) rDNA locus was performed on the Illumina MiSeq platform, using primers and protocols reported on the Nemabiome website (https://www.nemabiome.ca/) and previously described by Avramenko et al. [15], with slight modifications. Raw reads were cleaned by using Cutadapt version 3.4 [16], which finds and removes adapter sequences, primers, poly-A tails, and other types of unwanted sequences. The obtained reads were then analyzed by using the DADA2 R package version 1.12.1 [17], that implements a complete pipeline to turn paired-end fastq files from the sequencer into merged, denoised, chimera-free, inferred sample sequences. A 340 bp amplicon was obtained for all the seven samples and compared by BLAST analysis with publicly available databases, such as GenBank and the Nemabiome curated database. One of the obtained sequences was submitted to the European Nucleotide Archive (http://www.ebi.ac.uk/ena) under accession number ERZ4009650. One hundred percent coverage and identity with a *B. procyonis* sequence deposited in GenBank (accession number: MZ092853.1) was obtained for all seven samples.

Previous investigations on the parasitic fauna of raccoons in Italy did not highlight the presence of *B. procyonis* in the reproductive population set in Northern Italy [18], thus our study represents the first report of this parasite from Italy. Besides being the report of an alien species from a new country, this finding is worthy of being reported due to the potential sanitary relevance of this parasite for humans. All the infected raccoons originated from the same area of Casentino valley, Arezzo, Tuscany (Central Italy). The finding of this parasite in seven out of the 21 raccoons necropsied at present (33.3% prevalence) possibly suggests a high prevalence in the free-ranging population settled in Tuscany, with a transmission risk to people, wildlife, and domestic species [19]. This parasitosis could indeed affect both public health and native wildlife species susceptible to the transmission of the infection, since the raccoon population is settled near a national protected area, the Foreste Casentinesi, Monte Falterona e Campigna National Park.

Health risks associated with the introduction or translocation of alien vertebrate species into a new geographic area are well known. The vertebrate hosts have to be regarded as potential carriers of macro- and microparasites, agents of both animal and zoonotic diseases. The interaction of imported pathogens with new hosts (naïve or partially adapted) has often been the cause of serious consequences for animal and public health. Examples are diseases caused by the introduction of *Fascioloides magna*, rabies or African horse sickness viruses, *Mycobacterium bovis*, or *Brucella* spp*.* into a new biocenosis [20–23].

Although the risk of acquiring baylisascariasis is highest when synanthropic populations of raccoons dwell close to human settlings, or when raccoons are kept as pets [1, 2], *B. procyonis* eggs can remain infective for years in forest soil*,* and each infected raccoon can shed millions of eggs each day. Hence, the presence of infected raccoons in a natural area is likely to pose a public health risk [2], especially in a region like Casentino, which is geared towards ecotourism, hunting, and extensive farming. In Italy, the raccoon is regarded as an invasive species, and an eradication plan has been recently set up by official authorities [14]. The awareness of the presence in our country of its natural parasite *B. procyonis* should make the efforts aimed at its eradication even more stringent. The two populations of raccoon in Italy are not geographically contiguous and thus constitute two separate nuclei of different origin [18]*.* The Casentino raccoon population was first detected in 2013 [13], while the Lombardy population dates back to 2004. Suggested origins for the northern Italy population include dispersal from a feral raccoon population in Switzerland [12] or sporadic escapes or releases from private owners [24]. Since to the best of the authors’ knowledge the Northern Italy raccoon population is currently *B. procyonis-*free, every effort should be undertaken to avoid the merging of the two Italian reproductive populations, in order to prevent a further spread of this zoonotic parasite.

## Supplementary Information

**Additional file 1: Figure S1.** Numerous adults of *Baylisascaris procyonis* in the small intestine of an infected raccoon.

**Additional file 2: Figure S2.** Two female (top) and one male adults of *Baylisascaris procyonis*.

## Data Availability

One of the sequences obtained in this study is publicly available on the European Nucleotide Archive (ENA) under the accession number ERZ4009650. All other data generated or analyzed during this study are included in this published article.
