# Supplementary figures and images for: First report of the zoonotic nematode Baylisascaris procyonis in non-native raccoons (Procyon lotor) from Italy
Source: Parasit Vectors. 2022 Jan 12;15:24. doi: 10.1186/s13071-021-05116-3 (PMC8756652; doi:10.1186/s13071-021-05116-3)

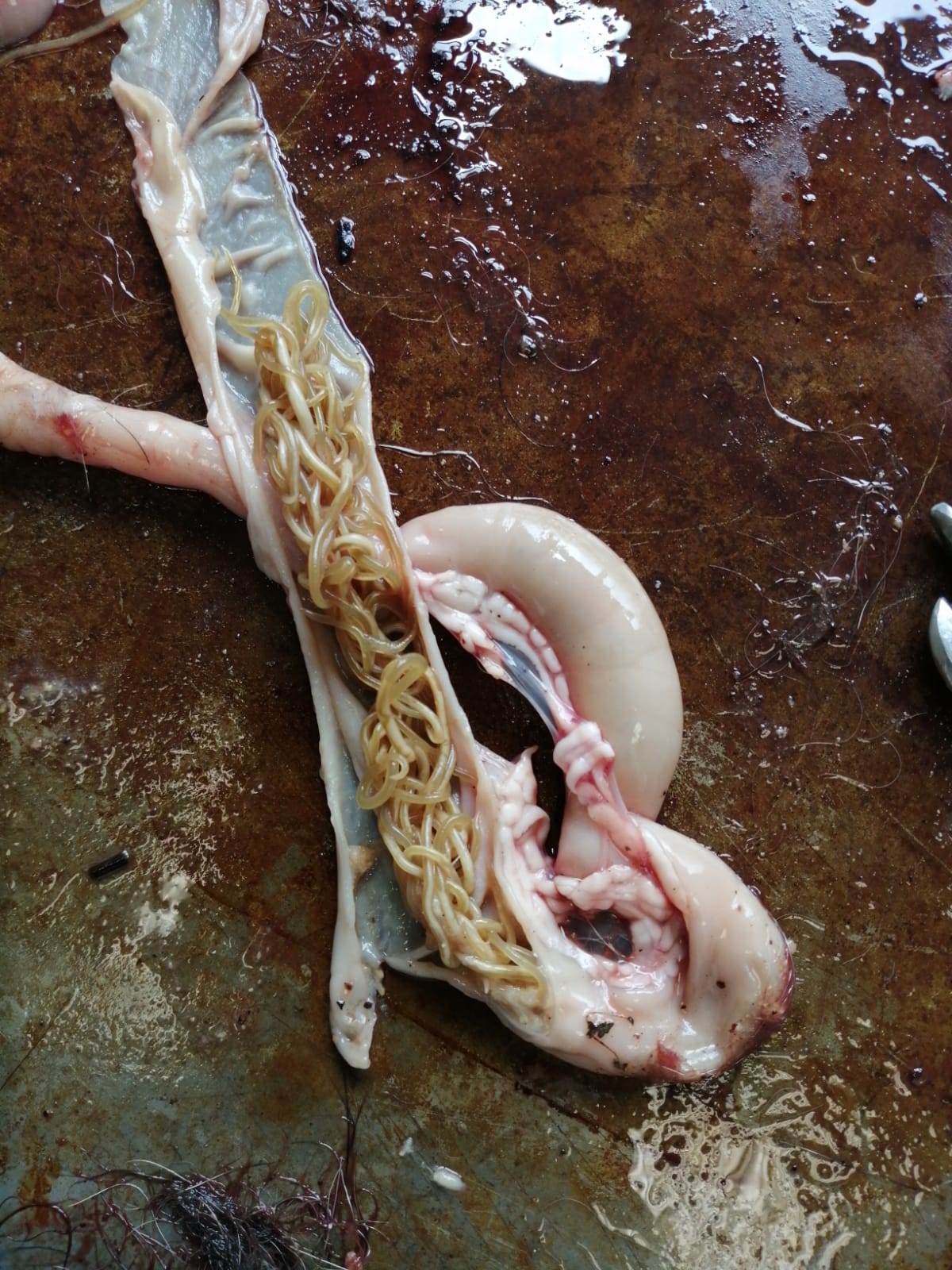

Supplement: Supplementary file 1 — Additional file 1: Figure S1. Numerous adults of Baylisascaris procyonis in the small intestine of an infected raccoon. [file 13071_2021_5116_MOESM1_ESM.jpg]

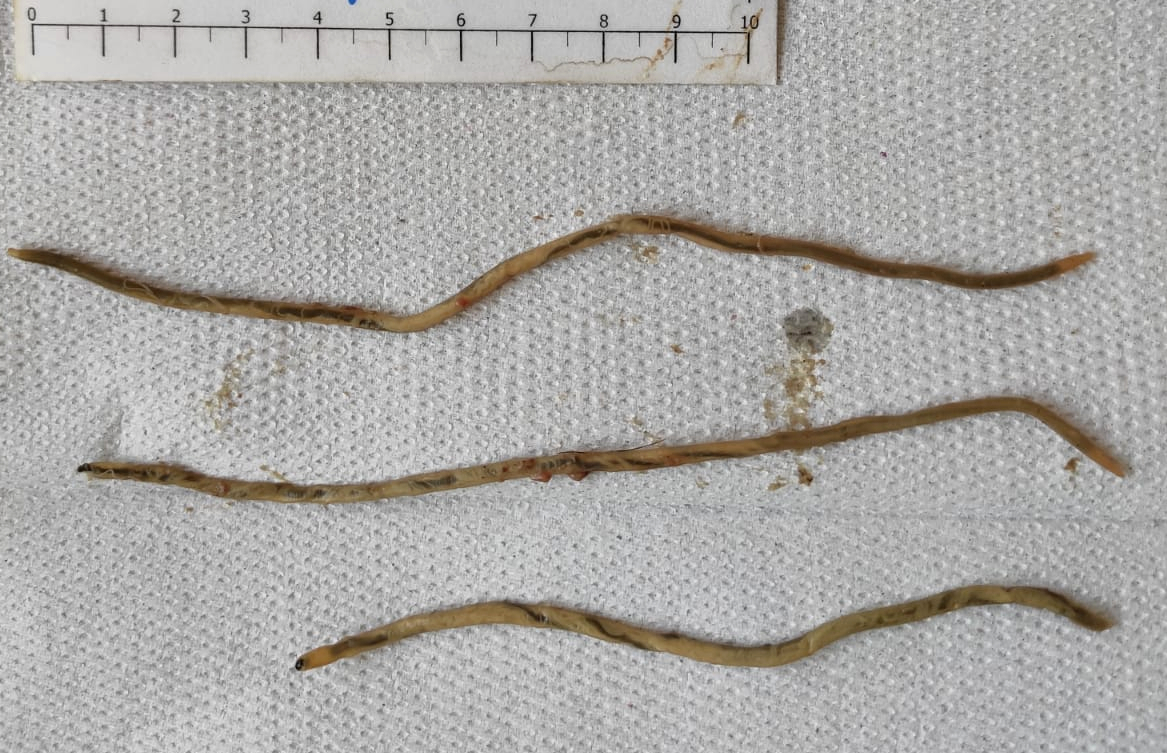

Supplement: Supplementary file 2 — Additional file 2: Figure S2. Two female (top) and one male adults of Baylisascaris procyonis. [file 13071_2021_5116_MOESM2_ESM.jpg]
